# Countering Gram-Negative Antibiotic Resistance: Recent Progress in Disrupting the Outer Membrane with Novel Therapeutics

**DOI:** 10.3390/antibiotics8040163

**Published:** 2019-09-24

**Authors:** Kelly M. Lehman, Marcin Grabowicz

**Affiliations:** 1 Microbiology and Molecular Genetics Program, Graduate Division of Biological and Biomedical Sciences, Laney Graduate School, Emory University, Atlanta, GA 30322, USA; kelly.marie.lehman@emory.edu; 2 Emory Antibiotic Resistance Center, Emory University School of Medicine, Atlanta, GA 30322, USA; 3 Department of Microbiology & Immunology, Emory University School of Medicine, Atlanta, GA 30322, USA; 4 Division of Infectious Diseases, Department of Medicine, Emory University School of Medicine, Atlanta, GA 30322, USA

**Keywords:** outer membrane, BamA, LptD, Bam, Lpt, Lol, arylomycin, globomycin

## Abstract

Gram-negative bacteria shield themselves from antibiotics by producing an outer membrane (OM) that forms a formidable permeability barrier. Multidrug resistance among these organisms is a particularly acute problem that is exacerbated by the OM. The poor penetrance of many available antibiotics prevents their clinical use, and efforts to discover novel classes of antibiotics against Gram-negative bacteria have been unsuccessful for almost 50 years. Recent insights into how the OM is built offer new hope. Several essential multiprotein molecular machines (Bam, Lpt, and Lol) work in concert to assemble the barrier and offer a swathe of new targets for novel therapeutic development. Murepavadin has been at the vanguard of these efforts, but its recently reported phase III clinical trial toxicity has tempered the anticipation of imminent new clinical options. Nonetheless, the many concerted efforts aimed at breaking down the OM barrier provide a source of ongoing optimism for what may soon come through the development pipeline. We will review the current state of drug development against the OM assembly targets, highlighting insightful new discovery approaches and strategies.

## 1. Introduction

As the incidence of multidrug-resistant (MDR) bacteria rises, the discovery of new classes of antibiotics is integral to the continuation of modern medical practices [1]. Gram-negative bacteria, in particular, are cause for alarm. Of the 14 pathogens listed as public health threats by the Centers for Disease Control and Prevention CDC, nine are Gram-negative [2]. Since the 1960s, new classes of antibiotics effective against these pathogens have proven elusive. A key cause of this failure is the presence of an outer membrane (OM) in Gram-negative bacteria. The OM is an essential organelle that has evolved to function as a permeability barrier, effectively excluding many current antibiotics and precluding their use in the clinic [3]. Moreover, the OM permeability barrier creates additional challenges for drug discovery. Given that an intact OM is required both for viability and for resistance against antibiotics, therapeutics targeting OM assembly have the potential both to kill bacteria outright and to sensitize them to antibiotics that are otherwise unable to penetrate an intact OM.

The composition of the OM is key to its function as a barrier. The lipid bilayer consists of an inner leaflet of phospholipids and an outer leaflet of the glycolipid lipopolysaccharide (LPS) [4]. Additionally, the OM contains an essential network of *β*-barrel outer membrane proteins (OMPs) and lipoproteins [5]. Strong lateral interactions between adjacent LPS molecules prevent hydrophobic and polar molecules from entering the bacterial cell [3]. Small hydrophilic molecules can enter the cell through OMPs that function as non-specific porins through the OM bilayer. However, such molecules are then often excreted through *β*-barrel or lipoprotein OM channels that are part of efflux pumps [6]. Together, the properties of the assembled OM create an impenetrable barrier to an array of antimicrobials. In addition, the inner membrane (IM) presents an orthogonal permeability profile; the IM strongly excludes small hydrophilic molecules while being permeable to small hydrophobic molecules. Together, the two membranes of the Gram-negative cell envelope present a daunting barrier against antibiotics with intracellular targets.

Gram-negative bacteria have evolved a series of machines required to navigate the challenges of building an OM. The proteins necessary for OM assembly are synthesized in the cytosol. After synthesis, the proteins must be translocated across the IM by the Sec translocon and transported across the aqueous periplasm to the OM [7]. Similarly, OM lipid molecules are all synthesized within the IM and must be delivered across the periplasm to the OM. There are three known pathways required for the transport of OM components: lipopolysaccharide transport (Lpt), *β*-barrel assembly machine (Bam), and the localization of lipoproteins (Lol) [7]. Lpt, Bam, and Lol transport highly hydrophobic materials across the aqueous periplasm: LPS [8], *β*-barrel proteins [9], and lipoproteins [10], respectively. How phospholipids reach the OM remains unclear [11].

Since the OM is essential for the cell, the function of the Lpt, Bam, and Lol pathways is also essential. These pathways are comprised of more than a dozen essential and highly conserved proteins that provide new targets for antimicrobials. No antibiotics in current clinical use target OM assembly, and no preexisting modes of resistance against these new targets are known or expected. The OM assembly machines were only recently discovered, and their functional mechanisms are still being explored. Nonetheless, the last decade has seen considerable effort devoted to finding lead molecules that disrupt OM assembly. The further development of these lead molecules could provide antibiotics active against the growing threat of multidrug-resistant Gram-negative bacteria.

Murepavadin, which targets the LPS transport system (discussed below), has thus far been the most promising antibiotic candidate that targets OM assembly. Murepavadin entered phase III trials. Unfortunately, the trials were recently suspended due to evidence of kidney toxicity. This disappointing milestone offers an opportunity to review the remarkable progress made in drug development targeting OM assembly. Many new and exciting compounds, both those which are directly active against OM assembly machines and those which prevent early and essential steps in OM biogenesis, have been discovered and offer renewed optimism for what may soon be coming through the development pipeline. We highlight the rare examples of compounds that already have demonstrated efficacy against clinical strains. The goal of this review is to emphasize the broad efforts targeting OM assembly and the discovery strategies that have proven successful in identifying OM-acting leads.

## 2. OMP Transport and Folding

**Figure 1:**
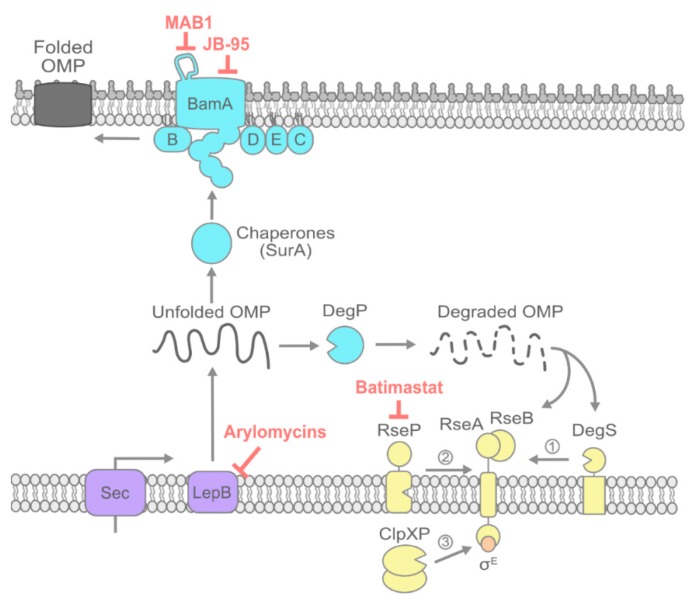
Outer membrane protein (OMP) biogenesis and its inhibitors. Targeted by an N-terminal signal peptide, OMPs are translocated to the inner membrane (IM) via the SecYEG translocon. After translocation, the signal peptide is cleaved by peptidases, such as LepB. Cleavage releases unfolded OMPs (uOMPs) into the periplasm, where they are transported to the outer membrane (OM) by chaperones. The BamABCDE complex receives, folds, and inserts OMPs at the OM. The periplasmic protease DegP degrades uOMPs in the periplasm if they accumulate or misfold. The *σ*^E^ stress response monitors OMP folding. Degraded uOMPs displace RseB from the anti-*σ* factor RseA, freeing RseA for cleavage by the protease DegS. Subsequent proteolysis by RseP and ClpXP releases *σ*^E^ into the cytosol to induce the transcription of stress regulon members. Red labels indicate compounds recently found to be active against steps of OMP biogenesis.

Gram-negative bacteria produce many OMPs that are critical to the cell. Some OMPs allow selective nutrient acquisition through the aqueous lumens of their *β*-barrel transmembrane domains [3,6]. Others have large surface-exposed domains attached to their *β*-barrels that interact with factors in the human host to support virulence [12]. Two OMPs, BamA and LptD, are essential in almost all Gram-negative bacteria, as they are necessary for OM biogenesis [13]. Therefore, targeting the assembly and proper folding of OMPs would stymie a variety of cellular functions. Moreover, the prevention of OMP folding causes an accumulation of unfolded intermediates in the periplasm that are acutely toxic to the cell. Therefore, OMP biogenesis, transport, and folding must be tightly controlled by the σ^E^ stress response. Altogether, OMP biogenesis offers several potential antibiotic targets (Figure 1).

### 2.1. Signal Peptide Processing by LepB

Nascent OMPs are translocated to the periplasm either co- or post-translationally [9]. A conserved N-terminal signal peptide targets OMP preproteins for translocation by the SecYEG translocon [14]. Once in the periplasm, OMPs are transiently tethered in the IM by their signal peptide, which must be cleaved before the mature protein can be transported to the OM. The signal peptidase I (SPase I), LepB in *E. coli*, is responsible for cleaving the signal peptide, releasing the mature OMP into the periplasm (Figure 1) [15]. With rare exceptions, LepB processes all OMPs and a majority of soluble periplasmic secreted proteins. The inhibition of LepB is the earliest opportunity to intercede in the OMP biogenesis pathway.

SPase I is essential in both Gram-negative and Gram-positive bacteria. The conserved Ser/Lys dyad active site of prokaryotic SPases is distinct from the active site of eukaryotic SPases [16]. Therefore, LepB is a promising target for the development of broad-spectrum antibiotics. The known SPase I inhibitors are part of a class of natural product lipoglycopeptides called arylomycins [17]. Crystallographic evidence suggests that arylomycin A_2_ mimics a signal peptide in its interaction with LepB, and likely occupies the catalytic site [18]. Early studies showed that arylomycins are active against Gram-positive bacteria, but have limited ability to permeate the OM of Gram-negative bacteria [17,19].

However, a recent study illustrates the power of optimizing natural scaffolds; using a crystal structure of an arylomycin bound to LepB, rational design was used to synthesize the derivative G0775 [20]. Unlike traditional arylomycins, G0775 can penetrate the OM, perhaps directly through the lipid bilayer, although this awaits clear confirmation. While a crystal structure of G0775 bound to LepB showed a binding site similar to traditional arylomycins, the modified molecule unexpectedly enabled an irreversible covalent interaction with the catalytic Lys residue [20]. This likely explains why G0775 is also markedly more potent against Gram-positive bacteria, because the SPase I target is freely accessible. G0775 is vastly more potent against a variety of Gram-negative pathogens, including MDR isolates, suggesting that the molecule is not subject to existing resistance mechanisms or efflux. Encouragingly, *lepB* mutations conferring resistance to G0775 are relatively infrequent [20]. In a thigh infection mouse model, G0775 was found to be effective against *E. coli, K. pneumoniae, P. aeruginosa,* and *Acinetobacter baumannii* [20]. Altogether, studies of G0775 have shown that it is an exciting potential therapeutic and a novel class of antibiotic active against Gram-negative bacteria.

### 2.2. Inhibiting OMP Chaperones

After cleavage of the signal peptide, highly hydrophobic unfolded OMPs (uOMPs) must cross the aqueous periplasm. The accumulation of uOMPs in the periplasm is toxic [21]; therefore, it is not surprising that several periplasmic chaperones rapidly transport OMPs to the Bam complex in the OM (SurA, Skp, DegP, FkpA) (Figure 1) [22,23,24]. Due to the redundancy of the chaperone network, none of the chaperones are individually essential for viability. However, different chaperones do appear to have specificity for particular uOMP substrates [23]. SurA is the main periplasmic chaperone of uOMPs and is important for the virulence of Gram-negative pathogens [25,26]. The essential OMP LptD is highly dependent on SurA for its biogenesis. The loss of *surA* reduces LptD levels and consequently impairs LPS transport; this causes OM permeability, which likely explains the attenuated virulence of Δ*surA* pathogens [27,28,29]. SurA may be a promising target for therapeutic discovery aimed at the prevention of infection. Indeed, in silico screening recently identified leads for in vivo testing [30].

### 2.3. Inhibiting the Bam Machine

Chaperones deliver OMPs to the Bam complex, which is responsible for their proper *β*-barrel folding and OM insertion [9]. Five proteins make up the complex (BamA–E). BamA is itself an OMP, while BamBCDE are all lipoproteins (Figure 1). BamA and BamD are both highly conserved and essential components of the BAM complex, making them ideal therapeutic targets [13,31]. BamD receives uOMPs from periplasmic chaperones, binding the “*β*-signal” peptide region at the C-terminus of uOMPs [32]. BamA, with the assistance of BamD, facilitates the folding of uOMPs into *β*-barrels [33,34,35]. The extracellular loops of BamA act as a dome, assisting in the folding of OMPs while also preventing solutes, such as antibiotics, from entering the cell [36,37,38]. The non-essential Bam lipoproteins (BamB, BamC, and BamE) likely assist Bam in accommodating a wide array of OMPs [39,40,41,42]. The crystal structures of the Bam complex have now been solved, opening new doors for the rational design of Bam-targeting drugs [9,36,37,38,43,44,45,46,47,48,49,50,51,52]. However, it is worth noting that the Bam complex seems to function via conformational cycling, and current structural information does not yet concretely demonstrate how Bam handles its client OMPs.

Few compounds have been identified that directly inhibit the Bam complex. A recent study identified monoclonal antibodies (mAbs) that inhibit BamA by screening mAbs from mice and rats immunized with *E. coli* and with purified *E. coli* BamA [53]. MAB1, one such BamA-binding antibody, inhibits BamA and is bactericidal against *E. coli*. MAB1 binds to extracellular loop 4 of BamA and appears to inhibit the ability of BamA to fold OMPs [53]. Inhibition of the folding of essential OMPs, such as LptD and nascent BamA, by MAB1 likely also causes cell death. However, the LPS of wild-type *E. coli* K-12 LPS prevents the binding of MAB1 to BamA. Therefore, MAB1 is only active against *E. coli* with deeply truncated LPS. Given that K-12 strains do not modify their LPS with the large O-antigen polysaccharides that are produced by all clinical *E. coli* strains and many other Gram-negative pathogens, more work will be required if any clinical potential of MAB1 is to be realized. Still, the discovery of MAB offers an exciting proof of concept: as mAbs cannot cross the OM, MAB1 proves that the function of BamA can be inhibited by an extracellular agent. Hence, MAB1 illustrates that the inhibition of BamA does not require a compound that can penetrate the OM, vastly expanding the chemical space in which to discover new inhibitors.

Another surface-exposed loop of BamA, loop 6, is critical for OMP folding, while conformationally cycling between exposed and buried states [54,55]. Loop 6 may represent a second high priority target for an extracellular inhibitor. The vulnerability of BamA to inhibition from outside of the cell seems to be unique among OM biogenesis factors. A recent comprehensive study of the surface-exposed loops of LptD (see later), and the antibodies against them suggests that functionally important LptD loops are not accessible to antibodies [56].

Additional efforts have highlighted the potential of targeting the Bam machine. For instance, engineering *E. coli* to secrete *β*-signal peptides that bind to BamD can interfere in the recognition of bona fide uOMPs. The effect is a cellular decrease in OMP levels, toxicity that impairs growth, and permeabilization of the OM to antibiotics [32]. This proof-of-concept study suggests that synthetic mimics of known *β*-signal peptides may prove to be potent therapeutics, especially as *β*-signals seem to be highly conserved [32,57].

In a separate study, a photoreactive derivative of the peptidomimetic antibiotic JB-95 caused the photolabeling of both BamA and LptD. Cells treated with JB-95 exhibited a decreased expression of several OMPs and an increased expression of OM stress response genes, suggesting a possible role in the inhibition of Bam [58]. JB-95, which was identified in a screen of peptides, also exhibits activity toward Gram-positive bacteria, demonstrating a lack of specificity for Bam. Since Bam activity is sensitive to lipid bilayer fluidity [53], one possibility is that JB-95 is a membrane-acting antibiotic. This may account for its observed effects on Bam and its activity against bacteria lacking Bam. Further studies of JB-95 are necessary to elucidate its primary target and mechanism of action.

### 2.4. Potentiating Bam Sensitivity by Inhibiting the σ^E^ Envelope Stress Response:

Defective Bam complex function causes uOMPs to accumulate in the periplasm, triggering activation of the *σ*^E^ extracytoplasmic stress response. The *σ*^E^ stress response mitigates the toxic effects of uOMP accumulation in a variety of ways, including downregulating the synthesis of new OMPs and upregulating the production of periplasmic proteases that degrade accumulated uOMPs [59]. The activation of σ^E^ requires multi-step proteolysis of the IM anti-σ factor RseA (Figure 1) [60]. Under steady-state growth, a periplasmic domain of RseA is bound by RseB, which prevents its cleavage by the protease DegS [61,62]. Envelope stress cues, such as the presence of degraded uOMPs in the periplasm, are sensed by both RseB and DegS. These cues cause the displacement of RseB from RseA, leaving RseA vulnerable to proteolysis by DegS [63,64,65,66,67]. The cleavage of the periplasmic domain of RseA enables the subsequent cleavage of RseA by RseP, which is an intramembrane zinc metalloprotease. RseP cleaves the RseA transmembrane domain between residues 108 and 109, releasing *σ*^E^ bound to a fragment of RseA into the cytosol [68,69]. The cytosolic protease ClpXP subsequently degrades the fragment, releasing *σ*^E^ and enabling it to activate transcription from cognate promoters [70].

To find inhibitors of OMP biogenesis, a recent screen exploited the remarkable properties of a *σ*^E^ gain-of-function mutation [21]. The screen compared the chemical sensitivity of two *E. coli* strains: one with wild-type *σ*^E^ and the other with a mutation in *σ*^E^ (encoded by *rpoES2R*) that primes—but does not activate—the stress response. The S2R substitution in RpoE enables a quicker and more robust response to OMP biogenesis defects and can suppress otherwise lethal mutations within the Bam complex [71]. In principle, *rpoES2R* should confer relative resistance (compared to wild-type *rpoE*) to chemical inhibitors specifically targeting OMP biogenesis, including those targeting Bam. Screening with this strategy identified batimastat [21], a known eukaryotic matrix metalloprotease inhibitor [72,73]. Curiously, batimastat caused a down-regulation of *σ*^E^ activity, which is essential in *E. coli* [21,74]. By priming the *σ*^E^ response, the S2R mutation conferred resistance against this *σ*^E^ inhibition by batimastat, satisfying the screening criteria.

Batimastat decreases *σ*^E^ activity by directly inhibiting RseP. The inhibition of RseP blocks the cleavage of RseA and prevents the subsequent steps toward the activation of *σ*^E^ [21]. The inhibition of *σ*^E^ activity by batimastat leads to an accumulation of uOMPs in the periplasm that proves to be lethal to *E. coli*. Indeed, overexpressing the periplasmic protease DegP to degrade the accumulated uOMPs is sufficient to confer batimastat resistance [21]. This finding provides reason to be optimistic that Bam inhibitors may be dual acting: depriving the OM of essential OMPs and generating toxic by-products in the periplasm that are bactericidal. Moreover, Batimastat provides a new approach to finding OMP biogenesis inhibitors, as blocking the *σ*^E^ response severely and specifically exacerbates defects in OMP biogenesis. Therefore, a Bam inhibitor used in conjunction with a σ^E^ inhibitor such as batimastat could be strongly synergistic.

## 3. LPS Transport

LPS is a unique bacterial glycolipid with properties that are perfectly suited for protecting the OM against both hydrophilic and hydrophobic antibiotics. LPS consists of a lipid A component that contains four to seven fully saturated acyl chains that tightly pack in the bilayer. A set of “core” oligosaccharides are attached to lipid A, and in many bacteria, the molecule is further decorated with an extended, charged, hydrophilic polysaccharide that can include more than 100 sugars. The core saccharides of individual LPS molecules each bear negative charges that enable bridging interactions to occur via divalent cation binding, which creates tight intermolecular interactions between adjacent LPS molecules.

Current clinically used OM-acting antibiotics directly disrupt LPS–LPS interactions at the OM. Polymyxins are the major family of such agents and remain in use as last-line antibiotics, despite their well-known nephrotoxicity [75]. Extensive research has been performed on the polymyxin family, and several reviews on their mechanism of action have been published [76,77,78]. Additionally, a number of LPS biosynthesis inhibitors have been in continued development [79]. However, the focus of this review is specifically on those antibiotics affecting OM assembly. The hope is that drugs blocking LPS transport to the OM might recapitulate the clinical efficacy of polymyxins while offering a more favorable toxicity profile.

### 3.1. Inhibiting Early Steps in LPS Assembly

**Figure 2:**
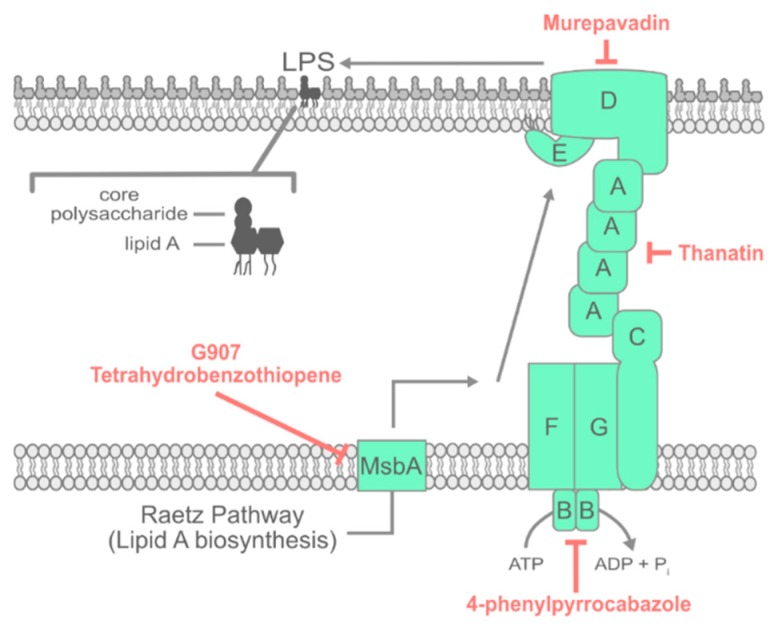
Lipopolysaccharide (LPS) biogenesis and its inhibitors. LPS is composed of lipid A, core polysaccharides, and the O antigen (not pictured). In the cytoplasm, lipid A is synthesized via the Raetz pathway. The flippase MsbA translocates LPS across the IM bilayer. The ATP-binding cassette (ABC) transporter LptB_2_FG extracts LPS molecules from the periplasmic leaflet of the IM. LPS travels from LptC, across an LptA bridge, and is received by LptD at the OM. LptDE facilitates the insertion of LPS into the OM. Red labels indicate compounds that are active against steps of LPS biogenesis and transport.

In order to fulfill its function in the OM, LPS must be synthesized and transported across the aqueous periplasm. The lipid A and core saccharide portions of LPS are synthesized at the cytoplasmic leaflet of the IM via the Raetz pathway (Figure 2) [80]; then, the molecules are translocated to the periplasmic leaflet of the bilayer by the flippase MsbA (Figure 2) [81,82]. Recently, two compounds that are active against MsbA were reported. A quinoline compound, G907, is an optimized and potent inhibitor of *E. coli* MsbA in vitro [83]. G907 prevents the ATPase activity of MsbA by binding the transmembrane pocket of the MsbA homodimer to lock the protein in a cytosol-facing LPS-bound state [83]. The combined effect is a potent inhibition of LPS flipping. G907 is active against *E. coli* and *K. pneumoniae* but shows reduced efficacy against *P. aeruginosa* [83].

A second MsbA-inhibiting compound was discovered contemporaneously using *Acinetobacter baylyi* and a clever genetic strategy [84]. Lipid A is non-essential in *A. baylyi*, and genes encoding early steps in LPS biosynthesis, such as *lpxA* (which encodes the first enzyme in the Raetz pathway of lipid A biosynthesis), can be readily deleted. Interestingly, the deletion of late-step biosynthetic genes or LPS transport *lpt* genes in *A. baylyi* is either impossible or poorly tolerated. This paradox arises due to a toxic accumulation of early LPS biosynthetic intermediates. Accordingly, in an Δ*lpxA* background, both late biosynthetic genes and *lpt* genes are freely dispensable.

Using the premise that the inhibition of Lpt transport should cause conditional toxicity, Zhang et al. designed a cell-based small molecule screen in which compounds were tested against two screening strains of *A. baylyi*, one with and one without functional early lipid A biosynthesis [84]. Using this screen, a hit molecule that inhibits late lipid A biosynthesis or LPS transport via Lpt would be toxic when lipid A is made, but would be non-toxic when early lipid A biosynthesis is inactivated by Δ*lpxA*. The screen was especially powerful, readily allowing the exclusion of off-target compounds, as lipid A-deficient or Lpt-defective cells are more permeable and more susceptible to a wide variety of chemicals. Through this elegant screening method, a tetrahydrobenzothiopene was identified, which directly targeted MsbA and caused the decoupling of ATP hydrolysis from actual LPS translocation [84]. The success of this screen illustrates how such strategies can be useful tools for the targeted discovery of compounds against an OM pathway. Indeed, an analogous strategy has also identified an inhibitor of the late LPS biosynthesis enzyme LpxK [85].

### 3.2. Inhibiting the Lpt Complex

From the IM, LPS must be transported across the periplasm via the seven-membered Lpt complex, LptA-G (Figure 2) [8]. LptB_2_FG forms an ATP-binding cassette (ABC) transporter which, in conjunction with LptC, remove LPS from the IM [86,87]. LptB hydrolyzes ATP in the cytosol [86,88], providing the power required to transport LPS across the periplasm, which has no available ATP [88]. LptA, a soluble periplasmic protein that multimerizes to form a bridge between the IM and OM, receives hydrophobic LPS molecules from LptC [89,90,91,92]. There is a constant flow of LPS from LptC to LptA and across the LptA bridge [88]. These data have informed the PEZ dispenser model of LPS transport in which sequential molecules of LPS moving from LptC to LptA physically push adjacent molecules of LPS further across the LptA bridge. At the OM, LptD and LptE receive LPS from LptA [93,94,95]. LptD is a *β*-barrel, and LptE is a lipoprotein that acts as a plug for LptD [96,97,98]. The N-terminal domain of LptD is homologous to LptA, providing a dock for the LptA bridge [88]. LPS enters the LptDE translocon through this N-terminal domain. Then, the LptDE translocon facilitates the insertion of LPS into the OM.

All seven members of the complex are essential for viability in organisms that require LPS, and the entire complex is broadly conserved [87,99]. The depletion of any members of the Lpt complex causes OM LPS deficiency and results in increased OM permeability [87] and OM defects [99]. It is worth noting that some species can survive without LPS in vitro and perhaps in immunopriveleged sites in vivo, but it is highly unlikely that LPS-deficient bacteria contribute significantly to clinical pathology in general. Thus, preventing the transport of LPS to the OM is a promising strategy for killing or increasing the permeability of Gram-negative bacteria.

The earliest identified OM assembly inhibitor was L27-11 [100], which was later renamed POL7080 and then murepavadin. This 14 amino acid synthetic peptidomimetic was based on protegrin I, which is a host-defense molecule previously shown to permeabilize membranes. Photoaffinity labeling suggests that the likely target of murepavadin is LptD. Mass spectrometry analysis demonstrated that treatment with murepavadin caused the cellular accumulation of LPS, suggesting the presence of defects in LPS transport consistent with LptD inhibition. Murepavadin is potently active against *P. aeruginosa*, but shows poor activity against other Gram-negative bacteria. This species-specificity is likely due to the extended N-terminal domain of the *P. aeruginosa* LptD homolog, which is not conserved in other species [100,101]. Murepavadin had formed the vanguard of future antibiotics targeting OM assembly, passing Phase I and Phase II trials. However, recent Phase III trials targeting adult nosocomial ventilator-associated *P. aeruginosa* pneumonia were suspended due to higher than expected kidney toxicity (NCT03582007 and NCT03409679). How this setback will affect murepavadin’s drive to the clinic is unclear at this time.

Compounds active against the Lpt intermembrane bridge have also been reported. Recent work described the antimicrobial peptide thanatin as an inhibitor of the Lpt complex [102]. Originally isolated from insects, thanatin is active against many Gram-negative bacterial species, including *E. coli* and *K. pneumoniae*. In a protein capture assay, thanatin was shown to interact with both LptA and LptD in vitro. Multiple thanatin-resistant mutants were isolated, with resistance mapping to LptA. A nuclear magnetic resonance (NMR) LptA–thanatin structure revealed the likely mechanism of action for thanatin: the inhibition of LptA complex formation with LptA, LptC, and LptD, all of which share a common OstA domain fold responsible for Lpt bridge formation. Although thanatin may prevent Lpt complex formation, the antimicrobial peptide is also active against Gram-positive bacteria, perhaps hinting at additional mechanisms of action that are, as of yet, unknown.

While the activity of several OM machines has now been reconstituted in vitro [103,104], a major challenge in the discovery of inhibitors is the scarcity of straightforward in vitro kinetic assays that are compatible with high-throughput screening approaches. One exception is an assay that is developed to measure LptB ATPase activity [105]. While screening kinase inhibitor libraries, two competitive inhibitors inactivating LptB were identified [106]. Analogs of one of the identified inhibitors, a 4-phenylpyrrolcabazole, were more potent inhibitors of LptB and of the LptB_2_FGC IM complex in vitro. ABC transporters are broadly conserved in biology, and achieving target specificity is critical. For example, the 4-pheylpyrrolcabazoles had already been developed as antagonists of a eukaryotic kinase target.

### 3.3. Activating Lpt Sensitizes Bacteria to Polymyxin

In addition to new drug discovery, the use of combinations of antibiotics is a possible alternate treatment strategy for Gram-negative pathogens. Recent work by the Ruiz and Kahne labs illustrated the power of drug synergy [107,108]. The antibacterial activity of the aminocoumarin antibiotic novobiocin is primarily due to its inhibition of DNA gyrase. The groups showed that novobiocin additionally stimulates LptB ATPase activity and LPS transport [107,108]. Although the stimulation of LPS transport alone is not toxic to bacteria, novobiocin treatment sensitizes *A. baumannii* to polymyxin [108]. As polymyxin antibiotics are often nephrotoxic at clinical concentrations, a combination of antibiotics that decreases the required dose of polymyxins could be a powerful clinical tool. It remains to be tested whether activated Lpt can re-sensitize clinical strains that are classified currently as polymyxin-resistant. Both the discovery of new drugs and the discovery of new therapeutic combinations of drugs will be integral to combatting resistance clinically.

## 4. Lipoprotein Transport

Lipoproteins are a family of triacylated secreted proteins. At least one essential OM lipoprotein is required for the function of both Bam and Lpt complexes [31,87,99]. Without lipoprotein trafficking, the OM of Gram-negative bacteria cannot be assembled. As it is central to OM biogenesis, the inhibition of lipoprotein trafficking could be an attractive target for antibiotics that can either kill Gram-negative bacteria or permeabilize them to antibiotics that otherwise cannot cross the OM. The lipoprotein trafficking pathway may be a particularly attractive drug target, since OM lipoproteins are essential components of Bam, Lpt, and even the Lol system itself. Fully exploiting this deep-seated dependency on lipoprotein trafficking could allow a single drug to deprive the cell of any new functional OM assembly machines.

### 4.1. Inhibiting Early Steps of Lipoprotein Maturation

**Figure 3:**
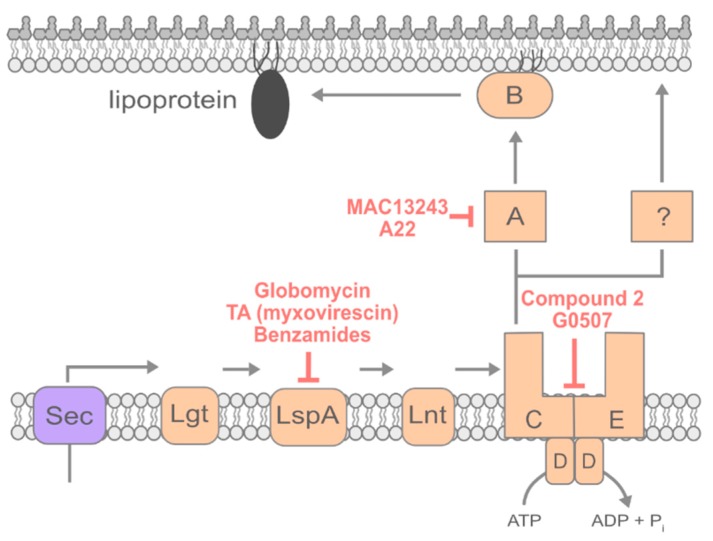
Lipoprotein trafficking and its inhibitors. Lipoproteins are synthesized in the cytoplasm and secreted by SecYEG. In the IM, lipoproteins undergo a series of modifications by Lgt, LspA, and Lnt to become mature triacylated species. The ATP-binding cassette (ABC) transporter LolCDE then extracts mature lipoproteins from the IM. LolA receives lipoproteins from LolC and traffics lipoproteins across the periplasm to the OM. At the OM, LolB receives and inserts lipoproteins. Recent work has indicated that an alternate route of lipoprotein trafficking must exist that does not require LolA or LolB. Red labels indicate compounds targeting lipoprotein maturation and trafficking. Lol: localization of lipoproteins.

Lipoproteins are made in the cytosol and translocated by Sec to the periplasm (Figure 3) [14,109]. Once secreted, lipoproteins remain anchored in the IM by their signal peptides. Then, lipoproteins are acylated and released from the signal peptide, after which most are trafficked to the OM [10,109]. The first modification to lipoproteins is the addition of diacylglycerol to an invariant cysteine residue in the lipobox by the IM enzyme Lgt [110]. LspA, a type II signal peptidase, then cleaves the signal sequence of lipoproteins at a conserved LAGC sequence. Subsequently, the diacylated cysteine becomes the first amino acid of the lipoprotein [111]. Then, the IM enzyme Lnt adds a third and final acyl chain to the amino group of the first cysteine, creating a triacylated lipoprotein ready to be trafficked to the OM [112].

Only fully mature, triacylated lipoproteins can be trafficked to the OM; thus, one possible strategy to prevent lipoprotein trafficking is the inhibition of lipoprotein maturation in the IM. Multiple inhibitors of LspA have been characterized [113,114]. By preventing the cleavage of the signal sequence, LspA inhibitors trap lipoproteins in the IM. In addition to preventing essential lipoproteins from reaching the OM, the mislocalization of some OM-targeted lipoproteins, such as Lpp in *E. coli* [115], has toxic consequences.

The compound globomycin, a hydrophobic cyclic peptide produced by *Streptomyces halstedii*, was identified in the 1970s and subsequently found to broadly inhibit LspA peptidases of both Gram-negative and Gram-positive species [116]. The crystal structure of globomycin bound to LspA was only recently solved, shedding light on the mechanism by which globomycin inhibits the signal peptidase [113]. The structure revealed that globomycin partitions into the membrane where it likely diffuses into the highly conserved active site of LspA. Globomycin mimics a signal peptide substrate and prevents access to the active site for true lipoprotein substrates, thereby impeding maturation and trafficking to the OM. 

Although the target of globomycin is clear, no resistant mutant variants of LspA have ever been recovered. The globomycin-bound LspA structure may provide a rationale for the lack of resistance. Globomycin forms an extensive interaction network with residues throughout the catalytic pocket, and it is perhaps unlikely that a single mutation could displace the antibiotic. It is also possible that mutations in these residues cause a substantial impairment to the activity of LspA. Globomycin is active against LspA in *Mycobacterium tuberculosis*, where this enzyme is non-essential [117]. Curiously, globomycin continues to exhibit bactericidal activity even in Δ*lspA M. tuberculosis* mutants, perhaps suggesting that globomycin may have additional targets and effects in the cell. Such additional activity could help further explain the lack of globomycin-resistant LspA mutants in *E. coli*.

Another macrocylic antibiotic that inhibits LspA is TA (myxovirescin), which is naturally produced by *Myxococcus xanthus* [114]. LspA overexpression in *E. coli* provides resistance against TA antibacterial activity. TA is more potent than globomycin against *E. coli*, but the permeability of both antibiotics across the OM is poor. The difficulty of the production and poor stability of both TA and globomycin suggest that neither will be used directly in the clinic. Nonetheless, globomycin and TA illustrate that LspA is a useful broad-spectrum antibiotic target.

A recent in vitro, FRET-based assay for LspA activity offers a step toward identifying specific LspA-acting molecules [118]. Using this assay, a large-scale chemical screen identified several leads, including a benzamide compound that could be optimized to yield potent LspA inhibitors [118]. Although these compounds were active against bacteria, they required the permeabilization of the OM to access LspA. Further work will be required to improve cell penetrance. However, LspA inhibitors that can be chemically synthesized will circumvent the difficulty associated with the isolation of natural LspA inhibitors. The introduction of a robust and high-throughput in vitro assay will likely enable the discovery of new chemical agents against LspA.

### 4.2. Inhibiting Lipoprotein Transport

Once a lipoprotein is fully acylated in the IM, it can be trafficked to the OM (Figure 3). In *E. coli*, the +2 residue is critical for the appropriate sorting of lipoproteins [119]. Lipoproteins with aspartate at +2 are retained in the IM, while alternate amino acids at the +2 residue allow for trafficking to the OM. Few exceptions to the IM retention rules have been described in *E. coli*, but sorting rules may vary in other organisms [10]. Those lipoproteins that do not have an IM sorting signal are trafficked to the OM by the ABC transporter LolCDE, which removes triacylated lipoproteins from the IM [120]. LolA, a periplasmic chaperone, receives lipoproteins from LolCDE and shields the highly hydrophobic acyl tails of lipoproteins as it traffics them across the aqueous periplasm [121]. LolB receives lipoproteins from LolA and inserts them into the OM [122]. All the Lol proteins are essential in wild-type bacteria. However, recent work identified a genetic background in which neither LolA nor LolB were required for viability or for lipoprotein trafficking in *E. coli*, suggesting that an alternate transport route must exist [123]. However, LolCDE remains absolutely essential to lipoprotein trafficking via either the LolAB or the alternate lipoprotein trafficking routes [123]. Thus, LolCDE represents a well-conserved target for therapeutic discovery. The inhibition of LolCDE will prevent the trafficking of essential lipoproteins to the OM while causing the toxic mislocalization of OM-targeted lipoproteins in the IM.

The first inhibitor of LolCDE, identified by Mcleod et al. in 2015, is called compound 2 [124]. Compound 2 is a pyridineimidazole and was identified in a phenotypic screen for *E. coli* growth inhibition of over 1.2 million compounds. *E. coli* and *Haemophilus influenzae* were both susceptible to compound 2. Unfortunately, no other Gram-negative bacteria tested showed susceptibility, despite the conservation of LolCDE. Mutations conferring resistance to compound 2 were isolated at the interface between the periplasmic and transmembrane regions of both LolC and LolE. Compound 2 was shown biochemically to inhibit the LolCDE-dependent release of lipoproteins from the IM.

Another inhibitor of LolCDE identified by Nayar et al. in 2015 was also called compound 2 [125]. The compound was identified using an AmpC *β*-lactamase reporter strain in which cell wall biogenesis defects cause increased production of AmpC that can be detected using nitrocefin, a colorimetric *β*-lactam substrate. Cell wall biogenesis defects were perhaps caused by compound 2 because of the impaired transport of LpoA and LpoB, which are two OM lipoproteins that are critical for cell wall biogenesis [126,127]. Mutants resistant to both compound 2 molecules were frequently isolated, and unsurprisingly given their shared chemical structures, cross-resistance to both compounds was observed [125].

**Figure 4:**
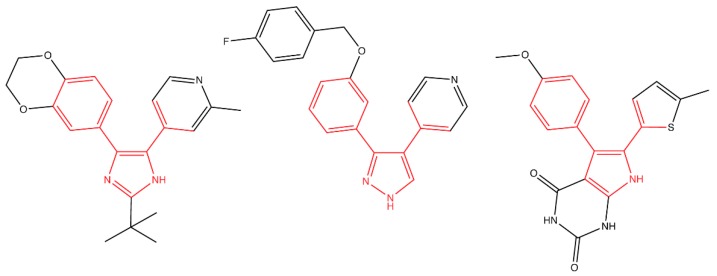
Inhibitors of the LolCDE complex show structural similarities. Nayar’s compound 2 (left), McLeod’s compound 2 (center), and Nickerson’s G0507 (right) all inhibit the LolCDE complex. Structural similarity between the three compounds is highlighted in red.

A screen of 35,000 synthetic molecules identified a third inhibitor of LolCDE, G0507 [128]. Nickerson et al. screened for compounds that both inhibited growth and caused up-regulation of the σ^E^ envelope stress response [128]. G0507 causes the accumulation of fully processed Lpp (the most abundant lipoprotein in *E. coli*) in the IM, indicating that lipoprotein trafficking is inhibited after processing [128]. G0507 causes the ATPase activity of the LolCDE complex to increase in vitro, which likely causes errant ATP hydrolysis [128]. Readily isolated point mutations in *lolC*, *lolD*, and *lolE* can all cause resistance to G0507. Interestingly, all three of the known LolCDE inhibitors share high structural similarity (Figure 4). Optimization of this core structure could potentially provide better LolCDE inhibitors. However, the frequent isolation of resistant mutants in LolC, LolD, and LolE indicates that discovering therapeutically useful inhibitors of LolCDE using this backbone may prove difficult.

LolA and LolB are also potentially interesting targets for novel therapeutics. The inhibition of these proteins should cause an accumulation of lipoproteins in the IM that would likely be toxic. Additionally, both LolA and LolB are essential in wild-type bacteria. A screen identified the compound MAC13243 as possibly targeting LolA [129]. MAC13243 kills *E. coli*, and this can be prevented by the heterologous overproduction of LolA. This phenotypic result, along with in vitro binding assays, seemed to suggest that MAC13243 directly inhibits LolA activity [129]. A later study found that MAC13243 degrades under aqueous conditions, becoming S-(4-chlorobenzyl)isothiourea [130]. This degradation product is a close analog of the compound A22—a known inhibitor of the essential actin homolog MreB, which spatially directs cell wall synthesis [130]. All three compounds (MAC13243, S-(4-chlorobenzyl) isothiourea, and A22) were found to have activity against Gram-negative bacteria, although the degradation products of MAC13243 had significantly more activity than MAC13243 itself [130]. The overexpression of LolA was found to increase resistance to A22, MAC13243, and S-(4-chlorobenzyl)isothiourea, while the depletion of LolA caused sensitization to the three compounds [130].

An independent screen illustrated that MAC13243 treatment caused increased cellular permeability in *E. coli*, which also occurs when LolA levels are depleted [131]. MAC13243 and A22 were suggested to act on LolA to permeabilize the OM. However, recent work from the Bernhardt group proposed an alternate explanation that could account for the resistance against A22, MAC13243, and related compounds conferred by LolA overproduction [132]. When LolA is produced in excess, the Rcs cell envelope stress response is activated, and LolA overproduction is only protective against the compounds when the Rcs response is intact. The Bernhardt group illustrated that inactivating RcsB, the transcriptional regulator of the Rcs system, abrogates the protective effect of LolA overproduction [132].

## 5. Conclusions

Recent research has illustrated that the essential machines required for OM biogenesis represent a bounty of potentially powerful therapeutic targets. Although few of the compounds that have been discovered so far are ready to be used clinically, the OM inhibitors identified to date have already provided mechanistic insights that will hopefully enable ever more innovative approaches to antibiotic discovery against Gram-negative bacteria. This knowledge base provides a starting point for optimization and offers new ideas for discovery efforts. Perhaps therapeutics can be designed based on optimization of the compounds discovered so far. For instance, arylomycins provide an interesting example of how careful optimization can drastically improve antibacterial efficacy.

It is notable that most of the lead inhibitors described in this review were identified using permeabilized strains, limiting their efficacy against clinical isolates with an intact OM and a full suite of efflux pumps. Compound optimization to facilitate improved penetrance and decreased efflux will be integral to identifying clinically useful therapeutics. Understanding the biochemical and biophysical properties that govern permeability across the OM remains a pressing goal. While notable progress has been made [133], generalizable and consistent rules for getting chemical agents into the Gram-negative cell envelope remain elusive, but such knowledge could empower entire generations of new drug discovery.
